# Dicyclohexyl(2′,4′,6′-triisopropylbiphenyl-2-yl)phosphine–dichlorophenylborane

**DOI:** 10.1107/S1600536809041865

**Published:** 2009-10-17

**Authors:** Holger Braunschweig, Rian D. Dewhurst, Krzysztof Radacki, Katharina Wagner

**Affiliations:** a Institut für Anorganische Chemie, Universität Würzburg, Am Hubland, D-97074 Würzburg, Germany

## Abstract

In the crystal structure of the title compound, C_39_H_54_BCl_2_P, the phospho­rus atom is coordinated by a dichloro­phenyl­borane unit. The substituted biphenyl group and the two cyclo­hexyl groups at the phospho­rus atom are arranged in such a way to avoid steric crowding in the mol­ecule as far as possible.

## Related literature

For related structures, see: Charmant *et al.* (2007 ▶); Grabulosa *et al.* (2005 ▶); Strieter *et al.* (2003 ▶).
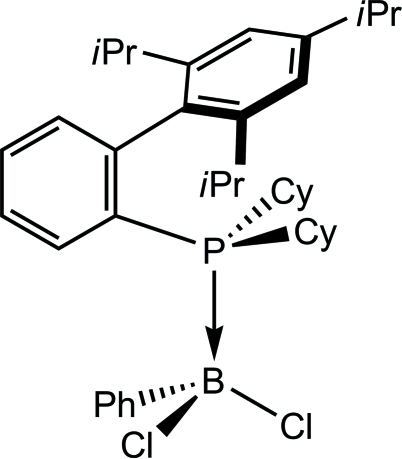


## Experimental

### 

#### Crystal data

C_39_H_54_BCl_2_P
*M* *_r_* = 635.50
Orthorhombic, 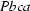

*a* = 16.9716 (8) Å
*b* = 15.5992 (7) Å
*c* = 26.5497 (12) Å
*V* = 7028.9 (6) Å^3^
*Z* = 8
Mo *K*α radiation
μ = 0.26 mm^−1^
*T* = 100 K
0.19 × 0.18 × 0.16 mm

#### Data collection

Bruker APEXII CCD diffractometer
Absorption correction: multi-scan (*SADABS*; Bruker, 2001 ▶) *T*_min_ = 0.844, *T*_max_ = 0.963
277684 measured reflections
10642 independent reflections
8513 reflections with *I* > 2σ(*I*)
*R*_int_ = 0.063

#### Refinement

*R*[*F* ^2^ > 2σ(*F* ^2^)] = 0.036
*wR*(*F* ^2^) = 0.088
*S* = 1.04
10642 reflections
394 parameters
H-atom parameters constrained
Δρ_max_ = 0.53 e Å^−3^
Δρ_min_ = −0.33 e Å^−3^

### 

Data collection: *APEX2* (Bruker, 2001 ▶); cell refinement: *SAINT-Plus* (Bruker, 2001 ▶); data reduction: *SAINT-Plus*; program(s) used to solve structure: *SHELXS97* (Sheldrick, 2008 ▶); program(s) used to refine structure: *SHELXL97* (Sheldrick, 2008 ▶); molecular graphics: *XP* in *SHELXTL* (Sheldrick, 2008 ▶); software used to prepare material for publication: *SHELXL97*.

## Supplementary Material

Crystal structure: contains datablocks I, global. DOI: 10.1107/S1600536809041865/bt5091sup1.cif

Structure factors: contains datablocks I. DOI: 10.1107/S1600536809041865/bt5091Isup2.hkl

Additional supplementary materials:  crystallographic information; 3D view; checkCIF report

## Figures and Tables

**Fig. 1.**
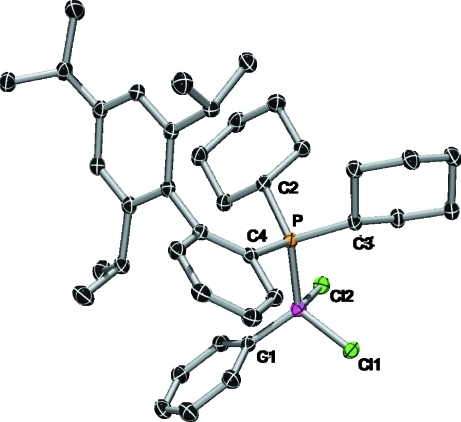
The molecular structure of (I). Displacement ellipsoids are drawn at the 50% probability level and H atoms have been omitted.

## supplementary crystallographic information

### Comment

The title compound, previously  not known in the literature, was prepared by the reaction of dichlorophenylborane with 2-dicyclohexylphosphino-2',4',6'-triisopropylbiphenyl. The second precursor is also known as XPhos and is used for example in Pd-catalysed C–N bond-forming processes (Strieter *et al.*, 2003). There is one similar structurally characterized compound (PhMe_2_PBCl_3_), which is a byproduct in the reaction of a platinum boryl complex with phosphine (Charmant *et al.*, 2007). However in this molecule there is no biphenyl ring attached to the phosphorus atom. The compound (2-biphenylyl)methoxyphenylphosphine-P-borane(1/1) which is used by Grabulosa *et al.* (2005) in the asymmetric hydrovinylation of styrene, contains a biphenyl ring but has no halogens at the boron. Beyond that this biphenyl ring is not substitued by isopropyl groups. So the choice of the substituents in the title compound makes it different from currently known compounds, in that the biphenylphosphines so far have not been combined with reactive haloboranes. The P—B distance is comparable with that found in PhMe_2_P—BCl_3_ (Charmant *et al.*, 2007) and in BiphMePhP—BH_3_ (Biph = Ph-2-Ph) (Grabulosa *et al.*, 2005) The B—Cl distance is comparable with those found in PhMe_2_PBCl_3_ (Grabulosa *et al.*, 2005). The biphenyl substituent is positioned away from the boron so that the isopropyl groups have sufficient space. The two phenyl rings of the biphenyl group adopt an almost perpendicular postition to each other, as is the typical conformation. The boron centre displays a slightly disordered tetrahedral coordination.

### Experimental

To prepare the title compound, 2-(dicyclohexylphosphino)-2',4',6'-triisopropylbiphenyl (0.20 g, 0.42 mmol) dissolved in 2 ml benzene, and dichlorophenylborane (0.07 g, 0.46 mmol) dissolved in 2 ml benzene were combined and stirred for 3 h at ambient temperature. All volatiles were removed under reduced pressure. The obtained solid was washed twice with 4 ml hexane. Recrystallization at ambient temperature from hexane gave colourless crystals of (I) 0.11 g (0.23 mmol, 54%).

### Crystal data

**Table:**

C_39_H_54_BCl_2_P	*F*(000) = 2736
*M**_r_* = 635.50	*D*_x_ = 1.201 Mg m^−^^3^
Orthorhombic, *P**b**c**a*	Mo *K*α radiation, λ = 0.71073 Å
Hall symbol: -P 2ac 2ab	Cell parameters from 9927 reflections
*a* = 16.9716 (8) Å	θ = 2.4–30.2°
*b* = 15.5992 (7) Å	µ = 0.26 mm^−^^1^
*c* = 26.5497 (12) Å	*T* = 100 K
*V* = 7028.9 (6) Å^3^	Block, colourless
*Z* = 8	0.19 × 0.18 × 0.16 mm

### Data collection

**Table:**

Bruker APEXII CCD diffractometer	10642 independent reflections
Radiation source: rotating anode	8513 reflections with *I* > 2σ(*I*)
multi-layer mirror	*R*_int_ = 0.063
φ and ω scans	θ_max_ = 30.5°, θ_min_ = 1.5°
Absorption correction: multi-scan (*SADABS*; Bruker, 2001)	*h* = −24→24
*T*_min_ = 0.844, *T*_max_ = 0.963	*k* = −22→20
277684 measured reflections	*l* = −37→37

### Refinement

**Table:**

Refinement on *F*^2^	Primary atom site location: structure-invariant direct methods
Least-squares matrix: full	Secondary atom site location: difference Fourier map
*R*[*F*^2^ > 2σ(*F*^2^)] = 0.036	Hydrogen site location: inferred from neighbouring sites
*wR*(*F*^2^) = 0.088	H-atom parameters constrained
*S* = 1.04	*w* = 1/[σ^2^(*F*_o_^2^) + (0.0317*P*)^2^ + 4.3765*P*] where *P* = (*F*_o_^2^ + 2*F*_c_^2^)/3
10642 reflections	(Δ/σ)_max_ = 0.003
394 parameters	Δρ_max_ = 0.53 e Å^−^^3^
0 restraints	Δρ_min_ = −0.33 e Å^−^^3^

### Special details

**Table:**

Geometry. All e.s.d.'s (except the e.s.d. in the dihedral angle between two l.s. planes) are estimated using the full covariance matrix. The cell e.s.d.'s are taken into account individually in the estimation of e.s.d.'s in distances, angles and torsion angles; correlations between e.s.d.'s in cell parameters are only used when they are defined by crystal symmetry. An approximate (isotropic) treatment of cell e.s.d.'s is used for estimating e.s.d.'s involving l.s. planes.
Refinement. Refinement of *F*^2^ against ALL reflections. The weighted *R*-factor *wR* and goodness of fit *S* are based on *F*^2^, conventional *R*-factors *R* are based on *F*, with *F* set to zero for negative *F*^2^. The threshold expression of *F*^2^ > σ(*F*^2^) is used only for calculating *R*-factors(gt) *etc*. and is not relevant to the choice of reflections for refinement. *R*-factors based on *F*^2^ are statistically about twice as large as those based on *F*, and *R*- factors based on ALL data will be even larger.

### Fractional atomic coordinates and isotropic or equivalent isotropic displacement parameters (Å^2^)

**Table:**

	*x*	*y*	*z*	*U*_iso_*/*U*_eq_	
B1	1.05541 (8)	0.09574 (8)	0.16307 (5)	0.0151 (2)	
Cl1	1.133928 (16)	0.082518 (18)	0.212825 (11)	0.01754 (6)	
Cl2	1.087313 (17)	0.033702 (18)	0.105927 (11)	0.01780 (6)	
P1	1.059876 (17)	0.220694 (19)	0.142349 (11)	0.01277 (6)	
C1_1	0.97152 (7)	0.06567 (7)	0.18435 (5)	0.0159 (2)	
C2_1	0.91983 (7)	0.01550 (8)	0.15579 (5)	0.0187 (2)	
H2_1	0.9337	0.0010	0.1222	0.022*	
C3_1	0.84859 (8)	−0.01383 (9)	0.17526 (5)	0.0227 (3)	
H3_1	0.8148	−0.0480	0.1550	0.027*	
C4_1	0.82682 (8)	0.00660 (9)	0.22411 (5)	0.0246 (3)	
H4_1	0.7783	−0.0134	0.2375	0.030*	
C5_1	0.87684 (8)	0.05679 (9)	0.25332 (5)	0.0241 (3)	
H5_1	0.8624	0.0714	0.2868	0.029*	
C6_1	0.94788 (7)	0.08555 (8)	0.23368 (5)	0.0202 (2)	
H6_1	0.9814	0.1196	0.2542	0.024*	
C1_2	1.02197 (7)	0.24348 (8)	0.07851 (4)	0.0153 (2)	
H1_2	1.0022	0.3039	0.0790	0.018*	
C2_2	1.08364 (7)	0.23841 (8)	0.03600 (4)	0.0181 (2)	
H2A_2	1.1285	0.2767	0.0439	0.022*	
H2B_2	1.1040	0.1791	0.0334	0.022*	
C3_2	1.04676 (8)	0.26500 (9)	−0.01429 (5)	0.0230 (3)	
H3A_2	1.0315	0.3262	−0.0126	0.028*	
H3B_2	1.0865	0.2588	−0.0414	0.028*	
C4_2	0.97440 (8)	0.21136 (9)	−0.02744 (5)	0.0245 (3)	
H4A_2	0.9904	0.1511	−0.0333	0.029*	
H4B_2	0.9503	0.2334	−0.0588	0.029*	
C5_2	0.91412 (7)	0.21498 (9)	0.01520 (5)	0.0223 (3)	
H5A_2	0.8690	0.1773	0.0070	0.027*	
H5B_2	0.8941	0.2743	0.0187	0.027*	
C6_2	0.95107 (7)	0.18630 (8)	0.06503 (4)	0.0176 (2)	
H6A_2	0.9686	0.1260	0.0622	0.021*	
H6B_2	0.9112	0.1896	0.0922	0.021*	
C1_3	1.16256 (7)	0.25864 (7)	0.14871 (5)	0.0151 (2)	
H1_3	1.1752	0.2516	0.1853	0.018*	
C2_3	1.17297 (7)	0.35500 (8)	0.13836 (5)	0.0171 (2)	
H2A_3	1.1614	0.3671	0.1025	0.021*	
H2B_3	1.1353	0.3880	0.1592	0.021*	
C3_3	1.25726 (7)	0.38311 (8)	0.15063 (5)	0.0194 (2)	
H3A_3	1.2641	0.4441	0.1414	0.023*	
H3B_3	1.2665	0.3776	0.1873	0.023*	
C4_3	1.31759 (7)	0.32892 (8)	0.12220 (5)	0.0198 (2)	
H4A_3	1.3109	0.3376	0.0855	0.024*	
H4B_3	1.3714	0.3475	0.1315	0.024*	
C5_3	1.30739 (7)	0.23414 (8)	0.13477 (5)	0.0199 (2)	
H5A_3	1.3166	0.2251	0.1712	0.024*	
H5B_3	1.3468	0.2000	0.1160	0.024*	
C6_3	1.22441 (7)	0.20354 (8)	0.12102 (5)	0.0182 (2)	
H6A_3	1.2181	0.1426	0.1307	0.022*	
H6B_3	1.2165	0.2082	0.0842	0.022*	
C1_4	1.00678 (7)	0.28522 (7)	0.18927 (4)	0.0138 (2)	
C2_4	1.04114 (7)	0.27716 (8)	0.23743 (4)	0.0164 (2)	
H2_4	1.0846	0.2394	0.2415	0.020*	
C3_4	1.01407 (7)	0.32198 (8)	0.27894 (5)	0.0185 (2)	
H3_4	1.0384	0.3147	0.3109	0.022*	
C4_4	0.95100 (7)	0.37757 (8)	0.27341 (5)	0.0181 (2)	
H4_4	0.9324	0.4100	0.3013	0.022*	
C5_4	0.91551 (7)	0.38515 (8)	0.22673 (5)	0.0179 (2)	
H5_4	0.8718	0.4228	0.2235	0.022*	
C6_4	0.94081 (7)	0.33994 (7)	0.18394 (4)	0.0144 (2)	
C1_5	0.89316 (7)	0.36063 (8)	0.13742 (4)	0.0149 (2)	
C2_5	0.91033 (7)	0.43574 (8)	0.11028 (5)	0.0160 (2)	
C3_5	0.86058 (7)	0.46083 (8)	0.07074 (5)	0.0176 (2)	
H3_5	0.8731	0.5110	0.0521	0.021*	
C4_5	0.79375 (7)	0.41455 (8)	0.05803 (4)	0.0172 (2)	
C5_5	0.77703 (7)	0.34126 (8)	0.08620 (5)	0.0173 (2)	
H5_5	0.7313	0.3090	0.0780	0.021*	
C6_5	0.82427 (7)	0.31315 (8)	0.12589 (5)	0.0159 (2)	
C1_6	0.98039 (7)	0.49241 (8)	0.12311 (5)	0.0180 (2)	
H1_6	1.0110	0.4637	0.1505	0.022*	
C2_6	1.03543 (7)	0.50442 (9)	0.07779 (5)	0.0229 (3)	
H2A_6	1.0518	0.4482	0.0651	0.034*	
H2B_6	1.0820	0.5370	0.0882	0.034*	
H2C_6	1.0076	0.5357	0.0512	0.034*	
C3_6	0.95346 (8)	0.58019 (9)	0.14270 (5)	0.0235 (3)	
H3A_6	0.9228	0.6094	0.1166	0.035*	
H3B_6	0.9997	0.6149	0.1513	0.035*	
H3C_6	0.9207	0.5723	0.1727	0.035*	
C1_7	0.73849 (7)	0.44011 (9)	0.01540 (5)	0.0197 (2)	
H1_7	0.7327	0.3893	−0.0073	0.024*	
C2_7	0.76810 (8)	0.51495 (11)	−0.01673 (6)	0.0323 (3)	
H2A_7	0.7708	0.5669	0.0040	0.048*	
H2B_7	0.7318	0.5244	−0.0449	0.048*	
H2C_7	0.8207	0.5015	−0.0298	0.048*	
C3_7	0.65651 (7)	0.46242 (9)	0.03553 (5)	0.0205 (2)	
H3A_7	0.6601	0.5126	0.0576	0.031*	
H3B_7	0.6356	0.4137	0.0546	0.031*	
H3C_7	0.6213	0.4752	0.0073	0.031*	
C1_8	0.79939 (7)	0.23518 (8)	0.15674 (5)	0.0183 (2)	
H1_8	0.8482	0.2065	0.1695	0.022*	
C2_8	0.75264 (8)	0.16926 (9)	0.12615 (6)	0.0254 (3)	
H2A_8	0.7013	0.1935	0.1170	0.038*	
H2B_8	0.7448	0.1174	0.1464	0.038*	
H2C_8	0.7819	0.1547	0.0955	0.038*	
C3_8	0.74970 (8)	0.26215 (9)	0.20256 (5)	0.0273 (3)	
H3A_8	0.7807	0.3005	0.2241	0.041*	
H3B_8	0.7345	0.2111	0.2218	0.041*	
H3C_8	0.7022	0.2920	0.1910	0.041*	

### Atomic displacement parameters (Å^2^)

**Table:**

	*U*^11^	*U*^22^	*U*^33^	*U*^12^	*U*^13^	*U*^23^
B1	0.0159 (6)	0.0135 (6)	0.0160 (6)	0.0009 (5)	−0.0015 (5)	0.0001 (5)
Cl1	0.01597 (13)	0.01586 (13)	0.02081 (14)	−0.00011 (10)	−0.00419 (10)	0.00321 (10)
Cl2	0.01872 (13)	0.01529 (13)	0.01940 (14)	0.00262 (10)	0.00104 (10)	−0.00148 (10)
P1	0.01143 (13)	0.01235 (13)	0.01451 (14)	0.00036 (10)	−0.00018 (10)	0.00016 (10)
C1_1	0.0148 (5)	0.0132 (5)	0.0197 (6)	0.0000 (4)	−0.0012 (4)	0.0026 (4)
C2_1	0.0180 (5)	0.0187 (6)	0.0195 (6)	−0.0015 (5)	−0.0024 (4)	0.0014 (5)
C3_1	0.0195 (6)	0.0212 (6)	0.0275 (7)	−0.0048 (5)	−0.0047 (5)	0.0034 (5)
C4_1	0.0178 (6)	0.0241 (7)	0.0320 (7)	−0.0035 (5)	0.0033 (5)	0.0055 (5)
C5_1	0.0242 (6)	0.0244 (6)	0.0237 (6)	−0.0015 (5)	0.0058 (5)	0.0004 (5)
C6_1	0.0211 (6)	0.0181 (6)	0.0213 (6)	−0.0021 (5)	0.0009 (5)	−0.0006 (5)
C1_2	0.0152 (5)	0.0152 (5)	0.0154 (5)	0.0018 (4)	−0.0010 (4)	0.0004 (4)
C2_2	0.0168 (5)	0.0215 (6)	0.0160 (6)	0.0005 (4)	0.0009 (4)	0.0006 (5)
C3_2	0.0232 (6)	0.0297 (7)	0.0162 (6)	0.0026 (5)	0.0009 (5)	0.0034 (5)
C4_2	0.0229 (6)	0.0337 (7)	0.0168 (6)	0.0048 (5)	−0.0032 (5)	−0.0015 (5)
C5_2	0.0184 (6)	0.0280 (7)	0.0206 (6)	0.0025 (5)	−0.0040 (5)	−0.0004 (5)
C6_2	0.0154 (5)	0.0197 (6)	0.0176 (6)	0.0004 (4)	−0.0012 (4)	−0.0007 (5)
C1_3	0.0122 (5)	0.0146 (5)	0.0185 (6)	−0.0010 (4)	−0.0006 (4)	0.0004 (4)
C2_3	0.0154 (5)	0.0149 (5)	0.0210 (6)	−0.0011 (4)	−0.0002 (4)	0.0009 (4)
C3_3	0.0170 (5)	0.0180 (6)	0.0232 (6)	−0.0041 (4)	0.0003 (5)	−0.0010 (5)
C4_3	0.0154 (5)	0.0225 (6)	0.0215 (6)	−0.0038 (5)	0.0017 (5)	0.0003 (5)
C5_3	0.0134 (5)	0.0224 (6)	0.0240 (6)	0.0004 (4)	0.0005 (4)	0.0016 (5)
C6_3	0.0132 (5)	0.0166 (6)	0.0248 (6)	0.0008 (4)	0.0009 (4)	0.0000 (5)
C1_4	0.0134 (5)	0.0121 (5)	0.0160 (5)	−0.0012 (4)	0.0009 (4)	0.0000 (4)
C2_4	0.0153 (5)	0.0156 (5)	0.0183 (6)	0.0001 (4)	−0.0012 (4)	0.0006 (4)
C3_4	0.0206 (6)	0.0189 (6)	0.0159 (5)	−0.0029 (5)	−0.0018 (4)	0.0000 (4)
C4_4	0.0218 (6)	0.0155 (6)	0.0171 (6)	−0.0019 (4)	0.0038 (4)	−0.0020 (4)
C5_4	0.0168 (5)	0.0157 (6)	0.0213 (6)	0.0022 (4)	0.0026 (4)	0.0004 (5)
C6_4	0.0130 (5)	0.0133 (5)	0.0170 (5)	−0.0015 (4)	0.0007 (4)	0.0014 (4)
C1_5	0.0133 (5)	0.0156 (5)	0.0158 (5)	0.0032 (4)	0.0016 (4)	0.0000 (4)
C2_5	0.0135 (5)	0.0160 (5)	0.0187 (6)	0.0023 (4)	0.0011 (4)	0.0004 (4)
C3_5	0.0155 (5)	0.0187 (6)	0.0185 (6)	0.0026 (4)	0.0018 (4)	0.0031 (5)
C4_5	0.0144 (5)	0.0206 (6)	0.0166 (5)	0.0045 (4)	0.0010 (4)	−0.0009 (4)
C5_5	0.0134 (5)	0.0189 (6)	0.0197 (6)	0.0013 (4)	−0.0004 (4)	−0.0018 (4)
C6_5	0.0134 (5)	0.0161 (5)	0.0184 (5)	0.0017 (4)	0.0017 (4)	−0.0010 (4)
C1_6	0.0149 (5)	0.0170 (6)	0.0220 (6)	−0.0008 (4)	−0.0013 (4)	0.0046 (5)
C2_6	0.0180 (6)	0.0204 (6)	0.0303 (7)	−0.0013 (5)	0.0037 (5)	0.0034 (5)
C3_6	0.0228 (6)	0.0225 (6)	0.0253 (6)	−0.0011 (5)	−0.0002 (5)	−0.0015 (5)
C1_7	0.0170 (6)	0.0264 (6)	0.0157 (5)	0.0029 (5)	−0.0018 (4)	0.0003 (5)
C2_7	0.0212 (6)	0.0502 (9)	0.0256 (7)	0.0002 (6)	−0.0007 (5)	0.0162 (7)
C3_7	0.0163 (5)	0.0253 (6)	0.0200 (6)	0.0027 (5)	−0.0006 (5)	0.0004 (5)
C1_8	0.0147 (5)	0.0163 (6)	0.0238 (6)	−0.0005 (4)	0.0011 (4)	0.0028 (5)
C2_8	0.0197 (6)	0.0200 (6)	0.0364 (7)	−0.0044 (5)	−0.0034 (5)	0.0016 (6)
C3_8	0.0273 (7)	0.0244 (7)	0.0302 (7)	−0.0008 (5)	0.0108 (6)	0.0039 (6)

### Geometric parameters (Å, °)

**Table:**

B1—C1_1	1.6020 (17)	C6_3—H6B_3	0.9900
B1—Cl2	1.8792 (14)	C1_4—C2_4	1.4108 (16)
B1—Cl1	1.8876 (13)	C1_4—C6_4	1.4151 (16)
B1—P1	2.0268 (13)	C2_4—C3_4	1.3836 (17)
P1—C1_4	1.8377 (12)	C2_4—H2_4	0.9500
P1—C1_2	1.8474 (12)	C3_4—C4_4	1.3854 (18)
P1—C1_3	1.8483 (11)	C3_4—H3_4	0.9500
C1_1—C2_1	1.3988 (17)	C4_4—C5_4	1.3828 (17)
C1_1—C6_1	1.4044 (17)	C4_4—H4_4	0.9500
C2_1—C3_1	1.3922 (17)	C5_4—C6_4	1.4044 (17)
C2_1—H2_1	0.9500	C5_4—H5_4	0.9500
C3_1—C4_1	1.386 (2)	C6_4—C1_5	1.5112 (16)
C3_1—H3_1	0.9500	C1_5—C2_5	1.4061 (17)
C4_1—C5_1	1.391 (2)	C1_5—C6_5	1.4174 (16)
C4_1—H4_1	0.9500	C2_5—C3_5	1.4029 (16)
C5_1—C6_1	1.3880 (18)	C2_5—C1_6	1.5202 (17)
C5_1—H5_1	0.9500	C3_5—C4_5	1.3862 (17)
C6_1—H6_1	0.9500	C3_5—H3_5	0.9500
C1_2—C6_2	1.5401 (16)	C4_5—C5_5	1.3954 (17)
C1_2—C2_2	1.5413 (16)	C4_5—C1_7	1.5230 (16)
C1_2—H1_2	1.0000	C5_5—C6_5	1.3947 (17)
C2_2—C3_2	1.5320 (17)	C5_5—H5_5	0.9500
C2_2—H2A_2	0.9900	C6_5—C1_8	1.5259 (17)
C2_2—H2B_2	0.9900	C1_6—C3_6	1.5345 (18)
C3_2—C4_2	1.5266 (19)	C1_6—C2_6	1.5346 (17)
C3_2—H3A_2	0.9900	C1_6—H1_6	1.0000
C3_2—H3B_2	0.9900	C2_6—H2A_6	0.9800
C4_2—C5_2	1.5269 (18)	C2_6—H2B_6	0.9800
C4_2—H4A_2	0.9900	C2_6—H2C_6	0.9800
C4_2—H4B_2	0.9900	C3_6—H3A_6	0.9800
C5_2—C6_2	1.5309 (17)	C3_6—H3B_6	0.9800
C5_2—H5A_2	0.9900	C3_6—H3C_6	0.9800
C5_2—H5B_2	0.9900	C1_7—C2_7	1.5306 (19)
C6_2—H6A_2	0.9900	C1_7—C3_7	1.5306 (17)
C6_2—H6B_2	0.9900	C1_7—H1_7	1.0000
C1_3—C2_3	1.5381 (16)	C2_7—H2A_7	0.9800
C1_3—C6_3	1.5431 (16)	C2_7—H2B_7	0.9800
C1_3—H1_3	1.0000	C2_7—H2C_7	0.9800
C2_3—C3_3	1.5314 (16)	C3_7—H3A_7	0.9800
C2_3—H2A_3	0.9900	C3_7—H3B_7	0.9800
C2_3—H2B_3	0.9900	C3_7—H3C_7	0.9800
C3_3—C4_3	1.5271 (17)	C1_8—C2_8	1.5317 (18)
C3_3—H3A_3	0.9900	C1_8—C3_8	1.5390 (18)
C3_3—H3B_3	0.9900	C1_8—H1_8	1.0000
C4_3—C5_3	1.5256 (18)	C2_8—H2A_8	0.9800
C4_3—H4A_3	0.9900	C2_8—H2B_8	0.9800
C4_3—H4B_3	0.9900	C2_8—H2C_8	0.9800
C5_3—C6_3	1.5313 (16)	C3_8—H3A_8	0.9800
C5_3—H5A_3	0.9900	C3_8—H3B_8	0.9800
C5_3—H5B_3	0.9900	C3_8—H3C_8	0.9800
C6_3—H6A_3	0.9900		
			
C1_1—B1—Cl2	112.95 (9)	C5_3—C6_3—C1_3	109.78 (10)
C1_1—B1—Cl1	110.41 (8)	C5_3—C6_3—H6A_3	109.7
Cl2—B1—Cl1	107.78 (7)	C1_3—C6_3—H6A_3	109.7
C1_1—B1—P1	114.24 (8)	C5_3—C6_3—H6B_3	109.7
Cl2—B1—P1	105.39 (6)	C1_3—C6_3—H6B_3	109.7
Cl1—B1—P1	105.58 (6)	H6A_3—C6_3—H6B_3	108.2
C1_4—P1—C1_2	110.23 (5)	C2_4—C1_4—C6_4	118.13 (10)
C1_4—P1—C1_3	103.00 (5)	C2_4—C1_4—P1	111.28 (8)
C1_2—P1—C1_3	110.52 (5)	C6_4—C1_4—P1	130.59 (9)
C1_4—P1—B1	108.93 (5)	C3_4—C2_4—C1_4	122.66 (11)
C1_2—P1—B1	114.90 (5)	C3_4—C2_4—H2_4	118.7
C1_3—P1—B1	108.57 (5)	C1_4—C2_4—H2_4	118.7
C2_1—C1_1—C6_1	116.73 (11)	C2_4—C3_4—C4_4	119.24 (11)
C2_1—C1_1—B1	122.01 (11)	C2_4—C3_4—H3_4	120.4
C6_1—C1_1—B1	121.20 (11)	C4_4—C3_4—H3_4	120.4
C3_1—C2_1—C1_1	121.82 (12)	C5_4—C4_4—C3_4	119.02 (11)
C3_1—C2_1—H2_1	119.1	C5_4—C4_4—H4_4	120.5
C1_1—C2_1—H2_1	119.1	C3_4—C4_4—H4_4	120.5
C4_1—C3_1—C2_1	120.23 (12)	C4_4—C5_4—C6_4	123.28 (11)
C4_1—C3_1—H3_1	119.9	C4_4—C5_4—H5_4	118.4
C2_1—C3_1—H3_1	119.9	C6_4—C5_4—H5_4	118.4
C3_1—C4_1—C5_1	119.24 (12)	C5_4—C6_4—C1_4	117.64 (11)
C3_1—C4_1—H4_1	120.4	C5_4—C6_4—C1_5	112.97 (10)
C5_1—C4_1—H4_1	120.4	C1_4—C6_4—C1_5	129.35 (10)
C6_1—C5_1—C4_1	120.18 (13)	C2_5—C1_5—C6_5	119.72 (11)
C6_1—C5_1—H5_1	119.9	C2_5—C1_5—C6_4	119.07 (10)
C4_1—C5_1—H5_1	119.9	C6_5—C1_5—C6_4	120.41 (10)
C5_1—C6_1—C1_1	121.81 (12)	C3_5—C2_5—C1_5	119.42 (11)
C5_1—C6_1—H6_1	119.1	C3_5—C2_5—C1_6	118.44 (11)
C1_1—C6_1—H6_1	119.1	C1_5—C2_5—C1_6	122.13 (10)
C6_2—C1_2—C2_2	109.31 (10)	C4_5—C3_5—C2_5	121.96 (11)
C6_2—C1_2—P1	111.96 (8)	C4_5—C3_5—H3_5	119.0
C2_2—C1_2—P1	115.18 (8)	C2_5—C3_5—H3_5	119.0
C6_2—C1_2—H1_2	106.6	C3_5—C4_5—C5_5	117.55 (11)
C2_2—C1_2—H1_2	106.6	C3_5—C4_5—C1_7	123.26 (11)
P1—C1_2—H1_2	106.6	C5_5—C4_5—C1_7	119.19 (11)
C3_2—C2_2—C1_2	110.30 (10)	C6_5—C5_5—C4_5	123.08 (11)
C3_2—C2_2—H2A_2	109.6	C6_5—C5_5—H5_5	118.5
C1_2—C2_2—H2A_2	109.6	C4_5—C5_5—H5_5	118.5
C3_2—C2_2—H2B_2	109.6	C5_5—C6_5—C1_5	118.23 (11)
C1_2—C2_2—H2B_2	109.6	C5_5—C6_5—C1_8	119.80 (11)
H2A_2—C2_2—H2B_2	108.1	C1_5—C6_5—C1_8	121.93 (11)
C4_2—C3_2—C2_2	112.31 (11)	C2_5—C1_6—C3_6	111.22 (10)
C4_2—C3_2—H3A_2	109.1	C2_5—C1_6—C2_6	111.80 (10)
C2_2—C3_2—H3A_2	109.1	C3_6—C1_6—C2_6	109.76 (10)
C4_2—C3_2—H3B_2	109.1	C2_5—C1_6—H1_6	108.0
C2_2—C3_2—H3B_2	109.1	C3_6—C1_6—H1_6	108.0
H3A_2—C3_2—H3B_2	107.9	C2_6—C1_6—H1_6	108.0
C3_2—C4_2—C5_2	110.44 (11)	C1_6—C2_6—H2A_6	109.5
C3_2—C4_2—H4A_2	109.6	C1_6—C2_6—H2B_6	109.5
C5_2—C4_2—H4A_2	109.6	H2A_6—C2_6—H2B_6	109.5
C3_2—C4_2—H4B_2	109.6	C1_6—C2_6—H2C_6	109.5
C5_2—C4_2—H4B_2	109.6	H2A_6—C2_6—H2C_6	109.5
H4A_2—C4_2—H4B_2	108.1	H2B_6—C2_6—H2C_6	109.5
C4_2—C5_2—C6_2	110.82 (10)	C1_6—C3_6—H3A_6	109.5
C4_2—C5_2—H5A_2	109.5	C1_6—C3_6—H3B_6	109.5
C6_2—C5_2—H5A_2	109.5	H3A_6—C3_6—H3B_6	109.5
C4_2—C5_2—H5B_2	109.5	C1_6—C3_6—H3C_6	109.5
C6_2—C5_2—H5B_2	109.5	H3A_6—C3_6—H3C_6	109.5
H5A_2—C5_2—H5B_2	108.1	H3B_6—C3_6—H3C_6	109.5
C5_2—C6_2—C1_2	110.59 (10)	C4_5—C1_7—C2_7	114.31 (11)
C5_2—C6_2—H6A_2	109.5	C4_5—C1_7—C3_7	111.08 (10)
C1_2—C6_2—H6A_2	109.5	C2_7—C1_7—C3_7	108.64 (11)
C5_2—C6_2—H6B_2	109.5	C4_5—C1_7—H1_7	107.5
C1_2—C6_2—H6B_2	109.5	C2_7—C1_7—H1_7	107.5
H6A_2—C6_2—H6B_2	108.1	C3_7—C1_7—H1_7	107.5
C2_3—C1_3—C6_3	112.40 (10)	C1_7—C2_7—H2A_7	109.5
C2_3—C1_3—P1	113.89 (8)	C1_7—C2_7—H2B_7	109.5
C6_3—C1_3—P1	114.80 (8)	H2A_7—C2_7—H2B_7	109.5
C2_3—C1_3—H1_3	104.8	C1_7—C2_7—H2C_7	109.5
C6_3—C1_3—H1_3	104.8	H2A_7—C2_7—H2C_7	109.5
P1—C1_3—H1_3	104.8	H2B_7—C2_7—H2C_7	109.5
C3_3—C2_3—C1_3	110.43 (10)	C1_7—C3_7—H3A_7	109.5
C3_3—C2_3—H2A_3	109.6	C1_7—C3_7—H3B_7	109.5
C1_3—C2_3—H2A_3	109.6	H3A_7—C3_7—H3B_7	109.5
C3_3—C2_3—H2B_3	109.6	C1_7—C3_7—H3C_7	109.5
C1_3—C2_3—H2B_3	109.6	H3A_7—C3_7—H3C_7	109.5
H2A_3—C2_3—H2B_3	108.1	H3B_7—C3_7—H3C_7	109.5
C4_3—C3_3—C2_3	111.27 (10)	C6_5—C1_8—C2_8	113.20 (11)
C4_3—C3_3—H3A_3	109.4	C6_5—C1_8—C3_8	110.98 (10)
C2_3—C3_3—H3A_3	109.4	C2_8—C1_8—C3_8	108.59 (10)
C4_3—C3_3—H3B_3	109.4	C6_5—C1_8—H1_8	108.0
C2_3—C3_3—H3B_3	109.4	C2_8—C1_8—H1_8	108.0
H3A_3—C3_3—H3B_3	108.0	C3_8—C1_8—H1_8	108.0
C5_3—C4_3—C3_3	110.62 (10)	C1_8—C2_8—H2A_8	109.5
C5_3—C4_3—H4A_3	109.5	C1_8—C2_8—H2B_8	109.5
C3_3—C4_3—H4A_3	109.5	H2A_8—C2_8—H2B_8	109.5
C5_3—C4_3—H4B_3	109.5	C1_8—C2_8—H2C_8	109.5
C3_3—C4_3—H4B_3	109.5	H2A_8—C2_8—H2C_8	109.5
H4A_3—C4_3—H4B_3	108.1	H2B_8—C2_8—H2C_8	109.5
C4_3—C5_3—C6_3	110.75 (10)	C1_8—C3_8—H3A_8	109.5
C4_3—C5_3—H5A_3	109.5	C1_8—C3_8—H3B_8	109.5
C6_3—C5_3—H5A_3	109.5	H3A_8—C3_8—H3B_8	109.5
C4_3—C5_3—H5B_3	109.5	C1_8—C3_8—H3C_8	109.5
C6_3—C5_3—H5B_3	109.5	H3A_8—C3_8—H3C_8	109.5
H5A_3—C5_3—H5B_3	108.1	H3B_8—C3_8—H3C_8	109.5
			
C1_1—B1—P1—C1_4	41.34 (10)	P1—C1_3—C6_3—C5_3	−172.51 (8)
Cl2—B1—P1—C1_4	165.91 (6)	C1_2—P1—C1_4—C2_4	−172.56 (8)
Cl1—B1—P1—C1_4	−80.17 (7)	C1_3—P1—C1_4—C2_4	−54.62 (9)
C1_1—B1—P1—C1_2	−82.88 (10)	B1—P1—C1_4—C2_4	60.51 (10)
Cl2—B1—P1—C1_2	41.69 (8)	C1_2—P1—C1_4—C6_4	6.24 (12)
Cl1—B1—P1—C1_2	155.62 (6)	C1_3—P1—C1_4—C6_4	124.18 (11)
C1_1—B1—P1—C1_3	152.81 (8)	B1—P1—C1_4—C6_4	−120.69 (11)
Cl2—B1—P1—C1_3	−82.62 (7)	C6_4—C1_4—C2_4—C3_4	−1.57 (17)
Cl1—B1—P1—C1_3	31.30 (8)	P1—C1_4—C2_4—C3_4	177.40 (10)
Cl2—B1—C1_1—C2_1	−15.69 (15)	C1_4—C2_4—C3_4—C4_4	−0.38 (18)
Cl1—B1—C1_1—C2_1	−136.45 (10)	C2_4—C3_4—C4_4—C5_4	1.60 (18)
P1—B1—C1_1—C2_1	104.75 (12)	C3_4—C4_4—C5_4—C6_4	−0.89 (19)
Cl2—B1—C1_1—C6_1	161.48 (10)	C4_4—C5_4—C6_4—C1_4	−1.07 (18)
Cl1—B1—C1_1—C6_1	40.73 (14)	C4_4—C5_4—C6_4—C1_5	−179.01 (11)
P1—B1—C1_1—C6_1	−78.08 (13)	C2_4—C1_4—C6_4—C5_4	2.22 (16)
C6_1—C1_1—C2_1—C3_1	−0.34 (18)	P1—C1_4—C6_4—C5_4	−176.51 (9)
B1—C1_1—C2_1—C3_1	176.96 (11)	C2_4—C1_4—C6_4—C1_5	179.77 (11)
C1_1—C2_1—C3_1—C4_1	0.21 (19)	P1—C1_4—C6_4—C1_5	1.03 (19)
C2_1—C3_1—C4_1—C5_1	0.1 (2)	C5_4—C6_4—C1_5—C2_5	80.69 (13)
C3_1—C4_1—C5_1—C6_1	−0.3 (2)	C1_4—C6_4—C1_5—C2_5	−96.95 (15)
C4_1—C5_1—C6_1—C1_1	0.2 (2)	C5_4—C6_4—C1_5—C6_5	−89.09 (13)
C2_1—C1_1—C6_1—C5_1	0.13 (18)	C1_4—C6_4—C1_5—C6_5	93.27 (15)
B1—C1_1—C6_1—C5_1	−177.19 (12)	C6_5—C1_5—C2_5—C3_5	−2.53 (17)
C1_4—P1—C1_2—C6_2	−87.52 (9)	C6_4—C1_5—C2_5—C3_5	−172.38 (11)
C1_3—P1—C1_2—C6_2	159.28 (8)	C6_5—C1_5—C2_5—C1_6	175.99 (11)
B1—P1—C1_2—C6_2	36.00 (10)	C6_4—C1_5—C2_5—C1_6	6.14 (17)
C1_4—P1—C1_2—C2_2	146.78 (9)	C1_5—C2_5—C3_5—C4_5	1.22 (18)
C1_3—P1—C1_2—C2_2	33.58 (10)	C1_6—C2_5—C3_5—C4_5	−177.35 (11)
B1—P1—C1_2—C2_2	−89.70 (10)	C2_5—C3_5—C4_5—C5_5	0.13 (18)
C6_2—C1_2—C2_2—C3_2	57.18 (13)	C2_5—C3_5—C4_5—C1_7	179.91 (11)
P1—C1_2—C2_2—C3_2	−175.76 (9)	C3_5—C4_5—C5_5—C6_5	−0.15 (18)
C1_2—C2_2—C3_2—C4_2	−56.22 (14)	C1_7—C4_5—C5_5—C6_5	−179.94 (11)
C2_2—C3_2—C4_2—C5_2	55.18 (15)	C4_5—C5_5—C6_5—C1_5	−1.15 (18)
C3_2—C4_2—C5_2—C6_2	−55.89 (15)	C4_5—C5_5—C6_5—C1_8	176.55 (11)
C4_2—C5_2—C6_2—C1_2	58.69 (14)	C2_5—C1_5—C6_5—C5_5	2.47 (17)
C2_2—C1_2—C6_2—C5_2	−58.93 (13)	C6_4—C1_5—C6_5—C5_5	172.19 (10)
P1—C1_2—C6_2—C5_2	172.21 (8)	C2_5—C1_5—C6_5—C1_8	−175.17 (11)
C1_4—P1—C1_3—C2_3	−59.75 (10)	C6_4—C1_5—C6_5—C1_8	−5.46 (17)
C1_2—P1—C1_3—C2_3	57.99 (10)	C3_5—C2_5—C1_6—C3_6	64.96 (14)
B1—P1—C1_3—C2_3	−175.14 (8)	C1_5—C2_5—C1_6—C3_6	−113.57 (13)
C1_4—P1—C1_3—C6_3	168.67 (9)	C3_5—C2_5—C1_6—C2_6	−58.12 (14)
C1_2—P1—C1_3—C6_3	−73.59 (10)	C1_5—C2_5—C1_6—C2_6	123.35 (12)
B1—P1—C1_3—C6_3	53.28 (10)	C3_5—C4_5—C1_7—C2_7	7.27 (18)
C6_3—C1_3—C2_3—C3_3	−54.13 (13)	C5_5—C4_5—C1_7—C2_7	−172.95 (12)
P1—C1_3—C2_3—C3_3	173.13 (8)	C3_5—C4_5—C1_7—C3_7	−116.10 (13)
C1_3—C2_3—C3_3—C4_3	54.90 (13)	C5_5—C4_5—C1_7—C3_7	63.68 (15)
C2_3—C3_3—C4_3—C5_3	−57.81 (14)	C5_5—C6_5—C1_8—C2_8	31.10 (16)
C3_3—C4_3—C5_3—C6_3	58.99 (14)	C1_5—C6_5—C1_8—C2_8	−151.28 (11)
C4_3—C5_3—C6_3—C1_3	−57.08 (14)	C5_5—C6_5—C1_8—C3_8	−91.30 (14)
C2_3—C1_3—C6_3—C5_3	55.20 (13)	C1_5—C6_5—C1_8—C3_8	86.31 (14)
